# C - reactive protein and interleukin - 6 levels among human immunodeficiency virus -infected patients with dysglycemia in Tanzania

**DOI:** 10.1186/s12902-019-0407-y

**Published:** 2019-07-22

**Authors:** Lilian Nkinda, Kirtika Patel, Benson Njuguna, Jean Pierre Ngangali, Peter Memiah, George M. Bwire, Mtebe V. Majigo, Mucho Mizinduko, Sonak D. Pastakia, Eligius Lyamuya

**Affiliations:** 1 Department of Microbiology and Immunology, Muhimbili University of Health and Allied Sciences, P.O box 65001, Dares Salaam, Tanzania; 2 Department of Immunology, Moi University, P.O. Box 4606-30100, Eldoret, Kenya; 3 Department of Pharmacy and Department of Cardiology, Moi Teaching and Referral Hospital, P.O Box 3-30100, Eldoret, Kenya; 4 Rwanda National Reference Laboratory, P.O Box 4668, Kigali, Rwanda; 5 Department of Public Health, University of West Florida, 11000 University Parkway, Pensacola, USA; 6 Department of Epidemiology and Biostatistics, Muhimbili University of Health and Allied Sciences, P.O Box 65001, Dares Salaam, Tanzania; 7 Purdue College of Pharmacy, Purdue Kenya Partnership, P.O Box 5760, Eldoret, Kenya

**Keywords:** CRP, HIV related inflammation, Diabetes, Pre-diabetes, Tanzania, Sub-Saharan Africa

## Abstract

**Background:**

Chronic inflammation has been associated with dysglycemia among people living with HIV (PLHIV). There is however, limited data regarding this phenomenon in sub-Sahara Africa (SSA). Therefore we assessed the levels of C-reactive protein **(**CRP) and Interleukin 6 (IL-6) on a cohort of PLHIV and its associations with dysglycemia in Tanzania.

**Methods:**

We conducted a cross-sectional study at the Infectious Disease Clinic (IDC) in Tanzania from March to May 2018. Purposive sampling was used to identify participants who had an undetectable viral load, were on 1st line anti-retroviral therapy (ART) and had an overnight fast. The WHO stepwise approach for non-communicable disease (NCD) surveillance was used to collect data. Fasting blood glucose and blood glucose after 75 g oral glucose load was measured, and Enzyme-linked immunosorbent assay (ELISA) was used to test for inflammatory markers (IL-6 and CRP). Associations were explored using the Chi square test and binary logistic regression was performed to estimate the odds ratios. A *p*-value less than 0.05 was considered statistically significant.

**Results:**

A total of 240 participants were enrolled. Forty two percent were overweight/obese (> 25 kg/m^2^), 89% had a high waist to height ratio. The median ART duration was 8(5–10) years. The prevalence of dysglycemia among our cohort of PLHIV was 32%. High CRP was associated with a 2.05 increased odds of having dysglycemia OR 2.05 (1.15–3.65) (*p* = 0.01). Taking stavudine was associated with a 1.99 odds of having dysglycemia OR 1.99 (1.04–3.82) (*p* = 0.03).We did not find a significant association between IL-6 and dysglycemia.

**Conclusion:**

High CRP and taking stavudine were significantly associated with dysglycemia among PLHIV with undetectable viral load.

## Background

Improved access to antiretroviral therapy (ART) and advances in HIV care in sub-Saharan Africa (SSA) has significantly increased life expectancy for people living with HIV (PLHIV) [1]. However, with aging and more time on ART, PLHIV are now more susceptible to non-communicable diseases (NCD) such as dysglycemia, that is, the presence of impaired fasting glucose (IFG), impaired glucose tolerance (IGT), or type 2 diabetes mellitus (T2DM) [2, 3]. Published reports from SSA reveal an escalating burden of dysglycemia among PLHIV [2, 4, 5], up to four times more compared to HIV negative controls [5]. This may be due to a mix of HIV related risk factors including chronic systemic inflammation [6, 7], use of ART such as stavudine [8], zidovudine [8], and protease inhibitors [4], and traditional risk factors such as physical inactivity, harmful alcohol use, smoking, overweight, and obesity [9–11].

It has been hypothesized that dysglycemia in PLHIV may be a result of an ongoing chronic inflammatory response [12, 13]. Several studies from high income countries (HIC) among PLHIV have demonstrated correlation between high levels of pro-inflammatory cytokines such as IL-6, CRP and tumour necrosis factor (TNF α), and dysglycemia, independent of body mass index (BMI) and age [12, 13]. In SSA, inflammation is reported to increase the risk of mortality [7, 14] and cardiovascular disease (CVD) among PLHIV [15], however, data on inflammation and dysglycemia is limited. Furthermore, extrapolating findings from HIC to PLHIV in SSA may not be reliable because of the existence of heterogeneity between inflammatory markers and type 2 diabetes based on race/ethnicity [16], a lower prevalence of traditional risk factors such as obesity in SSA compared to HIC which may influence systemic inflammation [17], and a higher background inflammatory state in the general SSA population [17]. SSA regional data on the association between chronic inflammation and dysglycemia among PLHIV is therefore needed.

The inflammatory markers of concern are many [18] but due to the biological and laboratory reproducibility of IL-6 and CRP, and their ability to predict the occurrence of non AIDS events [19], this study assessed the levels of CRP and IL-6 among PLHIV with dysglycemia in Tanzania.

## Methods

### Study site

We conducted this study at the Infectious Disease Clinic (IDC) at Ilala municipality in Dar es salaam, Tanzania. IDC operates a large care and treatment centre for PLHIV which attends to approximately 100 adult PLHIV per working day.

### Study design and study population

A cross-sectional study was carried out from March to May 2018. We included participants who were adult (above 18 years of age) PLHIV, had an overnight fast for at least 8 h, were currently on 1st line ART per applied guidelines in the setting [20], and had attained an undetectable viral load within the last 6 months. We excluded pregnant women, patients who had been on anti-inflammatory drugs within the last 3 months and those on 2nd line ART regimen including protease inhibitors (PI).

### Sample size and sampling technique

The sample size calculation was based on a reported prevalence of 17.4% of elevated CRP among patients with diabetes [18], and was calculated using Fisher’s formula. A minimum sample size of 220 was required to identify a prevalence of elevated CRP and IL-6 among HIV patients with dysglycemia, adjusted to 240 assuming a 10% non-response rate.

Purposive sampling was used for recruitment.

### Data collection

On enrolment, the WHO stepwise approach for NCD surveillance was used for data collection [21]. Additional information on CD4 count before ART initiation, after 6 months on ART, and the current CD4 count (within 6 months before study enrolment) was obtained from the files. The type of ART and duration on ART was also obtained from participants’ files. Demographic and behavioural characteristics such as age, sex, family history of diabetes, physical activity, and alcohol and smoking habits were collected through face to face interviews by a trained nurse counsellor. Physical measurements such as weight, height, and waist circumference were also taken by a trained nurse. Blood pressure was measured when a patient was seated and relaxed, using an *Omron M2 (*HEM-7121-E) automatic blood pressure device. Readings were taken two times consistently from the left arm, at three minutes interval.

### Cut off used for physical measurements

Waist to height ratio (WHR) was calculated, with a cut off of > 50% being considered to represent an elevated WHR [22]. BMI was calculated based on weight and height and the following cut-offs were used: under-weight (BMI < 18 kg/m^2)^, normal (BMI ≥ 18 kg/m^2^ and ≤ 25 kg/m^2^), overweight (BMI > 25 kg/m^2^and < 30 kg/m^2^, and obese (BMI ≥ 30 kg/m^2^). For ease of analysis, those who were overweight were combined with those who were obese, and those with normal BMI were combined with those who were underweight. Blood pressure was considered elevated if the average reading was ≥140/90 mmHg.

### Laboratory blood sample collection

Venous blood samples were taken for measurements, fasting and 2 h post 75 g oral glucose load from each participant. Serum was separated immediately after collection and measurements were done on the *Cobas-integra 400 + Roche* chemistry analyser within 30 min of collection. The remaining blood was stored in cryo vials at − 80 °C. The stored blood was used for IL-6 and CRP analysis using ELISA reader *Multiskan Ascent* reader 354–90593.

### Determination of dysglycemia

Dysglycemia was diagnosed by the presence of any of the following: i) impaired fasting glucose (IFG), defined as fasting blood glucose of 6.1 to 6.9 mmol/L and 2-h glucose < 7.8 mmol/L); ii) impaired glucose tolerance (IGT), defined as fasting blood glucose < 7.0 mmol/L and 2-h glucose ≥7.8 and < 11.1 mmol/L; and iii) diabetes mellitus (DM), defined as fasting blood glucose ≥7.0 mol/L or a glucose level ≥ 11.1 mol/l 2 h after a 75 g oral glucose load [5, 23].

### Laboratory procedure for IL-6 using ELISA

Indirect ELISA for IL-6 was performed using reagents from R&D systems, Catalogue number PD6050; we adopted their procedure as well. Briefly, a standard solution was prepared and serially diluted into five concentrations each half of the previous starting from concentrations of 300 pg/ml. Then 100 μl of sample, standard and controls were added into a pre-coated 96 well plate containing IL-6 monoclonal antibody in duplicates. Human IL-6 conjugate was added followed by a substrate solution after washing. Finally a stop solution was added changing the color of the well content into yellow from blue. Optical density was determined using a 450 nm and corrected by using a 630 wavelength.

A standard curve of mean absorbance of each standard against its concentration was plotted, the best fit line was determined by regression analysis. A y = mx + c equation was generated. The rest of the results were obtained by substituting y as the mean value of absorbance and calculating for X (concentration) for each patient result.

### Expected values

Concentration of IL-6 beyond 5 pg/ml was considered high [24].

### Laboratory procedure for CRP using ELISA

Indirect ELISA for CRP was performed using reagents from R&D systems, Catalogue number PD6050; we adopted their procedure as well. Briefly, a standard solution was prepared and serially diluted into five concentrations each half of the previous starting from a concentrations of 50 ng/ml, the samples were also diluted 100 folds (10 μl of sample+ 990 μl of diluents). Then 50 μl of sample, standard and controls were added into pre-coated 96 well plates containing CRP monoclonal antibody in duplicates. Human CRP conjugate was added followed by a substrate solution after washing. Finally a stop solution was added changing the color of the well content into yellow from blue. Optical density was determined using a 450 nm and corrected by using a 630 wavelength.

A standard curve of mean absorbance of each standard against its concentration was plotted, the best fit line was determined by regression analysis. A y = mx + c equation was generated. The rest of the results were obtained by substituting y as the mean value of absorbance and calculating for X (concentration) for each patient result. The concentration was then times by the dilution factor (100).

### Expected values

CRP concentration above 10 mg/litre was considered high [25]*.*

### Data analysis

Data from case report forms (CRFs) and ELISA reader were entered on an Excel spreadsheet and cleaned. Then data were exported to the statistical package for social sciences version 23 (SPSS software Chicago Inc., USA) for coding and statistical analysis. Descriptive statics were used to summarise participants’ characteristics. Bivariate analysis was used to look for association between the studied variables and the outcome measure. All variables with a *p* value ≤0.2 were subjected to a multi variate analysis with logistic regression. The magnitude of association was measured using adjusted odds ratio, and 95% confidence interval. A *p*-value < 0.05 was considered statistically significant.

## Results

### Participant characteristics

The characteristics of 240 participants who were enrolled and completed study procedures are summarised in Table 1. The mean age was 47 ± 10 years with the majority of participants being female (*n*=181[75%]). About 58(42%) participants were overweight/obese (> 25 kg/m^2^), 215(89%) had high waist to height ratio and 53(22%) had high systolic blood pressure. Ninety (37%) were involved in vigorous activities as part of their work and 110(46%) walked/cycled for more than 10 min to work for an average of 3 days a week.

**Table 1:** Participants’ characteristics

Variables	Value (*n* = 240)
Age (mean ± SD)	47 ± 10
Education level (%)	
No formal	29 (12%)
Primary	151(63%)
Secondary and above	60(25%)
Female	181(75%)
Family history of diabetes type 2	19(9%)
Current smoker (last 30 days)	5(3%)
Consumed alcohol in the last 30 days	36(15%)
Vigorous activity	90(37%)
Walking/bicycle (> 10 min)	110(46%)
BMI (%)	
Normal+Underweight	130(58%)
Overweight +Obese	58(42%)
Waist to height ratio (%)	
> 50%, (high)	215(89%)
High blood pressure (≥140/90 mmHg)	53(22%)
Baseline VL (Copies/ml) (%)	
0–999	210(93%)
1000+	15(7%)
Baseline CD4 (mean ± SD)	288 ± 231
CD 4 count after 6 months on ART(mean ± SD)	410 ± 223
Current CD4 (mean ± SD)	520 ± 236
Type or ART	
Stavudine based regimen	71(29%)
Zidovudine based regimen	86(35%)
Tenofovir based regimen	83(34%)
Efavirenz containing regimen	205(86%)
Median ART duration in years (IQR)	8(5–10)

The mean baseline CD4 count was 288cells/μl and 15(7%) had previously began ART treatment with a high viral load (1000+). After six months on ART, the mean CD4 count was 410cells/μl. The current CD4 count after a median ART duration of 8 years (IQR 5–10) was 520cells/μl. Seventy one (29%) were on a Stavudine (d4T) based regimen while 86(35%) were on a Zidovudine based regimen. All participants reported good adherence to ART.

### Sample prevalence of dysglycemia, CRP and IL-6

Among the study participants, 76(32%) had a dysglycemia, where 48(20%) had IFG, 26(11%) had IGT and 2(0.8%) had T2DM. No prior known diabetes cases were encountered during the study period. High CRP was observed among 80(33%) participants. Among participants with dysglycemia, high CRP was observed in 34(44%). High IL-6 levels were observed in 17(7%) participants. Among participants with dysglycemia, high IL-6 was observed in 7(9%) (Table 2).

**Table 2:** Prevalence of dysglycemia, CRP and IL-6

Variable	N (%)
Dysglycemia	76(32%)
Impaired fasting glucose (IFG)	48(20%)
Impaired glucose tolerance (IGT)	26(11%)
Diabetes mellitus type 2 (DM)	2(0.8%)
High CRP	80(33%)
Dysglycemia with High CRP	34(44%)
High IL-6	17(7%)
Dysglycemia with high IL-6	7(9%)

### Bivariate analysis

Among all the variables only CRP was significantly associated with dysglycemia (*p*-value of 0.01). Other variables with a *p*-value of < 0.2 included CRP level (*p*-value 0.01), systolic blood pressure (*p-*value 0.07), and being on a stavudine based regimen (*p*-value 0.06) were subjected to multivariate analysis. Results of bivariate analysis are shown in Table 3.

**Table 3:** Factors associated with dysglycemia in bivariate analysis

Variable	Dysglycemia N(%)	No dysglycemia N(%)	Chi square test (*p* value)
Age			
Below 20 years	1 (1.3)	1 (0.6)	1.958 (0.7)
20–29 years	2 (2.6)	8 (4.9)	
30–49 years	45 (57.7)	98 (60.5)	
50–64 years	25 (32.1)	49 (30.2)	
65–74 years	5 (6.4)	6 (3.7)	
Gender			
Male	23 (29.5)	36 (22.2)	1.499 (0.2)
Female	55 (70.5)	126 (77.8)	
BMI			
Normal + Underweight	44 (57)	96 (59)	0.175 (0.6)
Obese + Overweight	34 (43.5)	66 (41)	
Waist to height ratio			
< 50%, normal	8 (10.3)	17 (10.5)	0.003 (0.9)
> 50%, abnormal	70 (89.7)	145 (89.5)	
Blood pressure (≥ 140/90 mmHg)	22(28%)	31(19%)	2.5 (0.07)
CRP			
Low	44 (56.5)	116 (71.6)	5.470 (0.01)
High	34 (43.6)	46 (28.4)	
IL 6			
Low	71 (91)	152 (93.8)	0.628 (0.4)
High	7 (9)	10 (6.2)	
Current smoker			
No	77 (98.7)	153 (97.5)	0.364 (0.5)
Yes	1 (1.3)	4 (2.5)	
Consumed alcohol in the last 30 days			
No	65 (83.3)	139 (85.8)	0.252 (0.6)
Yes	13 (16.7)	23 (14.2)	
Vigorous activity			
No	50 (64.1)	100 (61.7)	0.127 (0.7)
Yes	28 (35.9)	62 (38.3)	
Parent Type 2 Diabetes			
No	72 (92.3)	148 (91.9)	0.011 (0.9)
Yes	6 (7.7)	13 (8.1)	
Stavudine containing regimen	17 (21.8)	54 (33.3)	3.365 (0.06)
Thymidine analogue (D4t or AZT)	49 (62.8)	108 (66.7)	0.344 (0.5)

### Multivariate analysis

Multivariate analysis was performed to adjust for potential confounders with *p* value< 0.2. While IL-6 did not meet this threshold, it was added in the adjusted model due to its potential to drive the CRP result [26]. On the multivariate analysis (Table 4), high CRP (> 10 mg/dl) OR 2.05 (1.15–3.65), (*p*-value 0.01) and taking stavudine OR 1.99 (1.04–3.82), (*p*-value 0.03) were significantly associated with dysglycemia.

**Table 4:** Factors associated with dysglycemia in Multivariate analysis

Variable	Un adjusted OR (95% CI)	*P* value	Adjusted OR (95% CI)	*P* value
CRP	1.95 (1.11–3.42)	0.02	2.05(1.15–3.65)	0.01
IL 6	1.49 (0.55–4.09)	0.43	1.2(0.45–3.68)	0.63
Blood pressure (≥140/90 mmHg)	1.7 (0.90–3.21)	0.11	1.69(0.88–3.24)	0.10
Stavudine based regimen	1.8(0.99–3.53)	0.05	1.99(1.04–3.82)	0.03

## Discussion

The prevalence of dysglycemia among our sampled PLHIV population on ART was 32%. We found that high CRP or being on a stavudine based regimen were significantly associated with having dysglycemia in multivariate analysis. A trend towards increased WHR (89%) and being overweight/obese BMI (44%), was observed among those with dysglycemia, but these did not reach statistical significance.

Although the prevalence of overt DM was low at 0.8%, the prevalence of pre-diabetes mellitus (pre-DM) comprising of IFG and IGT was high at 31.7%. In a recent review, the prevalence of DM and pre-DM was estimated to range from 1 to 26% and 19–47% among PLHIV in SSA [17]. The highest prevalence of DM and pre-DM was reported in Cameroon at 47 and 27% respectively among ART naive PLHIV, but no association with inflammatory markers was done [2]. In Tanzania, Maganga et al. found 18 and 14.7% DM and pre-DM prevalence amongst PLHIV respectively, however this burden could not be explained by known risk factors such as BMI, age, or ART duration among those receiving therapy, suggesting a potential role of inflammation [5]. Estimates from the HIV negative population in rural communities in northern Tanzania reported a comparatively lower prevalence of DM, and pre-DM, 2.8 and 2.5% respectively [27] which highlights the increased risk of dysglycemia among PLHIV. Furthermore, PrayGod et al. reported 1.5% and over 20% prevalence for DM and pre-DM respectively among underweight HIV-infected participants [28], and Mohammed et al. found a prevalence of 6.4 and 19.6% for DM and pre-DM respectively among Ethiopians [29]. While largely consistent, our study participants had longer times on ART (8 years vs 2 and 5 years) [28, 29]. The consistently high prevalence of pre-DM presents an opportunity for research on interventions to interrupt disease progression, as well as the urgent integration of routine screening for dysglycemia among PLHIV in SSA.

In our study, high CRP was associated with a 2 fold increased odds of having dysglycemia among PLHIV on 1st line ART with undetectable viral load. In SSA, studies on inflammation and dysglycemia among PLHIV are limited to a follow up cross sectional study [28] which did not find association between dysglycemia and either baseline or follow-up CRP after 2–3 years on ART. Inability to mount a CRP response could be explained by low CD4 count of 127 ± 99 μl/ml in their study as compared to 520 ± 236 μl/ml in the current study, as severe immunodeficiency (< 50 μl/ml) is reported to significantly limit inflammatory response, in addition to an increased risk of death [30]. This is observed in the above study [28] where only 57% out of 478 were alive at trial conclusion [28]. Several studies from HIC have reported higher levels of CRP and IL-6 to be associated with the incidence of dysglycemia [31, 32]. However how molecular mediators of inflammation lead to dysglycemia is not fully understood [33]. According to Lufti et al., immune inflammatory response may result to a change in metabolism by initiating insulin resistance in order to reduce energy consumption for body activities other than host defence [34]. Persistent inflammation among PLHIV due to inability to clear the virus or dysbiosis induce prolonged insulin resistance causing chronic restructure of metabolic pathways that may lead to diabetes [34, 35]. This is evidenced by a follow up study showing graded increases of high sensitive CRP (hs-CRP) and IL-6 to be associated with the increased risk of developing diabetes; those in the highest quartile of baseline plasma hsCRP had 5 times greater risk than those in the lowest quartile (HR = 5.13;95%CI 2.6–10.1), for those in the highest quartile of IL-6 rates were 3 times greater than those in the lowest quartile (HR 3.45;95% CI 1.91–6.23) [12]. Furthermore Brown et al. reported association between hs-CRP and incidence of diabetes at 48 weeks of ART initiation independent of known diabetes risk factors, however no significant associations with IL-6 were reported in this study [13]. These findings support chronic low-grade inflammation hypothesis in the development of DM, suggesting that CRP might be a useful marker in prediction of glucose abnormalities among PLHIV [28, 29], however, causation cannot be established through our current research design.

Of note, in this study, IL-6 had no association with dysglycemia. Weaker associations between dysglycemia and IL-6 compared to dysglycemia and CRP have also been observed in other studies [12, 36]. This may be because IL-6 has a much lower half life in circulation compared to CRP [37]. On the other hand, there are studies that reported IL-6 to be strongly associated to non AIDS events compared to CRP during HIV infection [38], therefore variability observed in our study may be due to chance. We did not find studies in SSA that looked at IL-6 as a potential risk factor for dysglycemia among PLHIV, and to the best of our knowledge, this is the first study that looked at both CRP and IL-6 and dysglycemia among SSA PLHIV.

Stavudine use was associated with a 1.99 fold risk of having dysglycemia. Twenty nine percent of our study participants were on this regimen despite WHO recommendations to discontinue the use of thymidine analogue NRTI [39]. Stavudine use has been consistently associated with insulin resistance and a 19% increased risk of developing DM [8, 40]. The proposed mechanism involves inhibition of enzyme DNA polymerase-γ responsible for mitochondrial function [8]. This results to mitochondrial dysfunction that leads to insulin resistance [41]. Studies conducted among PLHIV have been complicated by the confounding effect of HIV infection where a reduction of mitochondrial DNA is observed in absence of ART [42]. However data from HIV uninfected populations have demonstrated a 52% reduction of muscle mitochondrial DNA after 1 month of stavudine exposure [43]. Therefore consumption of stavudine for PLHIV may exacerbate mitochondrial dysfunction, hastening the occurrence of diabetes. The risk is ever increasing with cumulative exposure. This was not observed in our study with a median ART duration of 8 years. Abrahams et al. [44] also did not find association with dysglycemia in PLHIV with a median duration of use of 6.8 years on stavudine. On the contrary, a 9 year follow up study in Senegal that involved participants who received stavudine among other regimens reported a 10% increased risk of dysglycemia after 4 years of treatment [45]. The majority of studies that have reported a lack of association between ART consumption and dysglycemia have had either a small sample size or a small number of different ART combinations [5].The overwhelming benefits of ART outweigh the occurrence of non AIDS events [46], therefore efforts to counteract the adverse events may be more useful to reduce the burden of co-morbidities.

Traditional risk factors for dysglycemia in PLHIV include: older age [4, 47], male gender [9, 48], family history of diabetes [49], higher BMI [9, 50], high WHR [51], smoking [52], alcohol consumption [52], hypertension [29] and physical inactivity [28]. We did not find a statistically significant association for any of the above with dysglycemia in this study. Our study participants were majority female (75%), reported low prevalence of smoking (3%), alcohol consumption (15%), and family history of DM (9%), which may also have limited our ability to find associations between these risk factors and dysglycemia. There was a trend towards increased WHR (89%) and being overweight/obese BMI (42%) among those with dysglycemia, but this did not reach statistical significance. Participants with dysglycemia were slightly younger with the majority falling in the 30–49 age category, however the association was also not statistically significant.

While our findings are among the few studies on inflammation and dysglycemia in Africa, they remain correlative. Research gaps that can be explored in the future include: i) conducting a longitudinal study that will further assess the relationship between inflammation and incident dysglycemia, and ii)intervention studies among the pre-DM PLHIV population to prevent disease progression to DM. Different interrupters can be explored such as nutrition, anti-inflammatory agents and lifestyle modifications.

One of the main limitations of this study was that 75% of study participants were female. It was challenging to get men to participate, possibly because of the 2 h wait period for the OGTT test. Poor male participation in health services is reported in several studies in Africa and this has been associated with a higher mortality among HIV positive males compared to females in SSA [53]. A small sample size may have also limited the ability to detect other significant associations. Finally, our study did not use high sensitive CRP (hs-CRP) to measure CRP, which may have underestimated the burden of inflammation.

## Conclusion

This study showed high CRP was significantly associated with dysglycemia among PLHIV with undetectable viral load. Taking stavudine was associated with increased odds of dysglycemia.

## Data Availability

All data used to draw conclusion of the study is provided in the manuscript.

## Abbreviations

ART: Antiretroviral therapy
BMI: Body mass index
CRFs: Case report forms
EACEA: Education, audiovisual and culture executive agency
HIC: High income countries
Hs-CRP: High sensitive C reactive protein
IL-6: Interlukin-6
MTRH: Moi teaching and referral hospital
NACP: National AIDS control program
NCD: Non communicable disease
NRTI: Nucleoside reverse transcriptase inhibitors
OGTT: Oral glucose tolerance test
PLHIV: People living with HIV
SSA: Sub Sahara Africa
T2DM: Type 2 diabetes mellitus
TNF: Tumour necrosis factor
WHR: Waist height ratio
